# Characterization of Failure Behavior in Unidirectional Fiber-Reinforced Polymer via Off-Axis Compression on Small Block Specimens

**DOI:** 10.3390/polym15244699

**Published:** 2023-12-13

**Authors:** Fan Yang, Yazhi Li, Biao Li

**Affiliations:** Department of Aeronautical Structural Engineering, School of Aeronautics, Northwestern Polytechnical University, Xi’an 710072, China; tonyyoung789@mail.nwpu.edu.cn

**Keywords:** fiber-reinforced polymer (FRP), off-axis compression, failure criteria, fiber-kinking theory

## Abstract

An experimental investigation was focused on the failure behavior of unidirectional fiber-reinforced polymers when subjected to combined longitudinal/transverse compression and in-plane shear due to off-axis loading. Block-shaped and end-loaded specimens, spanning ten different fiber orientations (0°, 5°, 10°, 15°, 20°, 30°, 45°, 60°, 75°, and 90° with respect to the loading direction), were loaded to ultimate failure using a dedicated fixture. Different failure modes, including longitudinal compression, in-plane shear, and transverse compression, were identified, along with distinctive characteristics of the corresponding failure envelopes. Four physically based failure theories—Hashin, Camanho, Puck, and LaRC05—were subjected to a comparative analysis. Criteria derived from the concept of the action plane consistently outperformed in describing matrix-dominated failures, providing both qualitative and quantitative predictions of failure stresses and fracture plane orientation. However, for fiber-dominated failures, these theories seem to fall short in providing satisfactory predictions, particularly in accurately describing the influence of shear on fiber compression failure. Although criteria based on fiber-kinking theory can reasonably explain the formation of kink bands, they tend to yield overly conservative results. Recalibrations and minor refinement based on experimental results were implemented, leading to an improved agreement. Finally, the constructive role of off-axis compression tests in characterizing the failure behavior of unidirectional composites is discussed.

## 1. Introduction

Over the past few decades, the application of fiber-reinforced polymers (FRPs) in lightweight structures has been boosted, particularly in aerospace, aviation, and automobile industries, as well as other cutting-edge fields. This, in turn, has spurred structural engineers to explore a wider spectrum of design possibilities. Unidirectional laminates of FRPs, typically seen as homogenous and transversely isotropic, are often used to evaluate the mechanical performance of composite material systems, which is a prerequisite for fully exploiting their potential in structural applications. However, the inherent heterogeneity and anisotropy introduce significant complexity, particularly with respect to the diversity of failure mechanisms. The material response and failure behavior in the principal directions have been thoroughly understood, and the associated properties are commonly determined using established standards. However, relying on this information alone is not sufficient to ensure the reliability and optimization of composite structural designs, which necessitates time-consuming and costly testing, often accompanied by the retention of excessive safety margins.

Considerable research efforts have been devoted to the failure modes and envelopes of unidirectional FRPs, giving rise to a variety of failure theories and criteria. However, the proliferation of these theories has confused researchers and engineers, as there is no definitive guidance on how to select an appropriate theory for specific materials or application scenarios. To alleviate this confusion, several evaluations have been launched since the turn of the century. A noteworthy endeavor is the series of World-Wide Failure Exercises (WWFEs) [1,2], which are international collaborative activities that have made a remarkable contribution to enhancing comprehension of the latest advances in failure theories. They have brought to light significant disparities among existing failure theories under biaxial or multiaxial loading, especially when the coupling between compression and shear is involved. Meanwhile, the acquisition of reliable test data under combined loading conditions has received much attention, and the paucity of such data has brought considerable obstacles to validation work. On the other hand, to accurately delineate the failure envelope, an increasing amount of research has been devoted to elucidating various failure phenomena. With this growing understanding, more sophisticated criteria have been developed that incorporate not only basic strength properties (such as tensile and compressive strength along or perpendicular to the fiber, in-plane shear strength, etc.), but also additional non-standard parameters, preferably calibrated from biaxial or multiaxial test data.

Various multiaxial test methods for composites and their respective specimen configurations have been reviewed in the literature [3]. Multiaxial stress states in fiber-reinforced composites are generally achieved in two main ways. Internal multiaxiality results from the anisotropic behavior of the material, while external multiaxiality occurs when loads are applied in different directions, as seen in cruciform or tube specimens. Cruciform specimens [4], developed from previous experience with similar metal specimens, provide the flexibility to perform tests under almost arbitrary biaxial (in-plane) loading. Special attention should be paid to corner fillets and reduced thicknesses to ensure that failure occurs within the gauge section. In some cases, design optimization of the specimen configuration may be essential prior to formal testing. Tubular specimens [5,6] are another option that eliminates the problems associated with free edge effects. They allow for a wide range of biaxial and triaxial stresses through combinations of internal or external pressure, torsion, and axial loading. However, their representativeness for typical laminated planar structures is often questioned due to differences in manufacturing processes. There are also other multiaxial loading schemes, such as a custom biaxial testing facility for combined in-plane shear and normal stresses [7]. What all of these tests have in common is that they require a sophisticated testing machine, with two or more actuators, and a control system with precise feedback loops, often in conjunction with complex specimen-holding devices. As a result, while some of these testing methods have been proposed for some time, they have remained predominantly in the realm of academic research and pose challenges for practical implementation in engineering practice.

The off-axis testing of unidirectional laminates provides a balanced alternative to complex loading tests. By adjusting the angle between the fiber direction and the loading direction, a certain range of biaxial stress states can be obtained under uniaxial loading. The versatility of off-axis testing has been demonstrated in a number of studies to investigate the constitutive and failure behavior of fiber-reinforced composites [8,9,10]. Nevertheless, applied loads that do not coincide with the predominant orientation of the material can cause additional shear deformation, resulting in a non-uniform strain field. Consequently, while many researchers have employed off-axis testing to analyze failure mechanisms, fewer have used it directly to determine strength parameters.

This situation changes greatly when block-shaped specimens are used and compressive loads are applied through the end faces. As long as the loading ends of the specimen keep laterally unconstrained, maximum uniformity of strain and stress throughout the specimen is ensured. Bing and Sun [11] studied the specimen size effect in off-axis compression tests and concluded that it is negligible when the contact friction on the loading surface is effectively eliminated. Ma et al. [12] installed a fixture for the loading of small bulk specimens and conducted experimental studies on the failure mechanism of unidirectional AS4/PEEK, a carbon-fiber-reinforced thermoplastic composite. The same specimen form is also applicable for dynamic testing using split Hopkinson pressure bars. Tsai and Sun [13] performed both quasi-static and dynamic experiments with 15°, 30°, and 45° off-axis block specimens to study the in-plane shear strength and failure strain of unidirectional glass–epoxy S2/8552. Koerber et al. [14] conducted similar experiments on carbon–epoxy IM7/8552, employing advanced observation techniques such as high-speed photography and digital image correlation. Moreover, thanks to the specimen size and loading method, the off-axis compression test based on small block specimens is ideally suited for environmental testing under varying temperature and humidity conditions, as well as for real-time observation in an electron microscope chamber.

This paper investigates the failure behavior of unidirectional carbon/epoxy laminates under off-axis compression. The ultimate strengths and failure modes under combined longitudinal/transverse compression and in-plane shear are compared with the predicted results from prevailing failure theories: Hashin [15,16], Comanho [17], Puck [18], and LaRC05/Pinho [19,20]. Recalibration and simple modifications based on the experimental results were also made, leading to an improved agreement. In addition, the positive role of off-axis compression tests in characterizing the failure behavior of unidirectional composites is discussed.

## 2. Off-Axis Compression Test

### 2.1. Material and Specimens

The carbon–epoxy composite system selected for experimental investigations is CCF300/BA9916-II, which is composed of intermediate modulus fibers and interlayer-toughened epoxy resin, with a fiber volume fraction of 60%. Several fundamental mechanical properties have been measured using standardized testing methods and are listed in Table 1. All the preliminary measurements of material properties and the subsequent off-axis compression tests were conducted on the same batch of laminates.

As generally recommended, the in-plane shear strength is to be determined from the truncated point of the stress–strain curve at 5% engineering shear strain, since ultimate failure occurs well beyond this strain, where fiber rotation becomes pronounced. In fact, measuring in-plane shear strength is a difficult task and, so far, there seems to be no ideal testing method that can guarantee a pure and uniform in-plane shear stress state up to failure. This issue will be discussed in more detail later.

**Figure 1:**
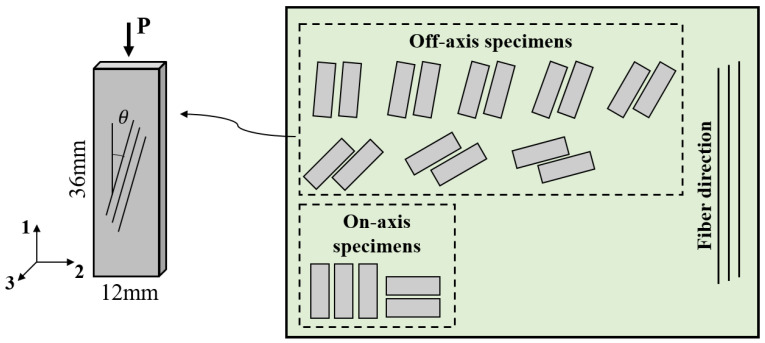
Preparation and geometry of specimens.

All specimens were fabricated from 24-ply unidirectional laminates with an average thickness of 3 mm. As illustrated in Figure 1, small blocks were cut at different angles with respect to the fiber direction using a water-cooled diamond saw. The nominal dimensions are 36 mm in length and 12 mm in width, resulting in an in-plane aspect ratio of 3 to mitigate the end effect. To obtain different failure modes and typical features of the failure envelope, up to eight off-axis angles—5°, 10°, 15°, 20°, 30°, 45°, 60°, 75°—were selected, along with two special angles—0° and 90—corresponding to longitudinal and transverse compression, respectively. Six samples were prepared for each group to ensure repeatability. Smooth contact surfaces are essential to allow shear-induced deformation to take place freely under off-axis compression. Hence, the ends of all specimens were first polished with sand papers. The parallelism tolerances of all opposing surfaces were strictly controlled so that both ends could be in close contact with the loading surfaces during the test.

### 2.2. Testing Procedures

All compressive specimens were subjected to monotonic loading until failure on a DDL100 electronic universal load frame (Sinotest Equipment Co, Ltd., Changchun, China) under an ambient environment. Displacement control was applied with a stroke speed of 0.1–0.2 mm/min, which equates to a strain rate of less than 10${}^{-4}$/s. Slower speeds were utilized for specimens with off-axis angles close to 0°, as the stiffness along the fiber direction is basically an order of magnitude larger than that perpendicular to the fiber.

**Figure 2:**
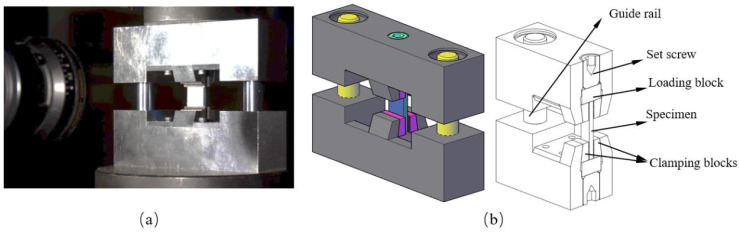
Test set-ups: (**a**) load frame; (**b**) fixture.

To ensure the alignment of loading plates and eliminate eccentricity, a specialized fixture was designed and fabricated, as shown in Figure 2. The specimen was positioned between the upper and lower parts of the fixture, which could move steadily to each other along two guide columns. Low friction and smooth movement are guaranteed by employing clearance-free guide units with high-precision linear bushings, with a selected shaft diameter of 12 mm. Stainless steel loading blocks with surface hardening were used to prevent damage to the loading surfaces due to the high compressive strength of the carbon fiber. Each end of the specimen was inserted into a clamping region fitted with a pair of wedged clamping blocks, leaving approximately 12 mm of space for observation. During mounting, lateral supporting force from the wedged clamping blocks could be applied by adjusting the set screw, which assists in centering the specimen and prevents it from tripping over or moving accidentally. As mentioned above, over-restraints introduced by clamping force ought to be released before testing. There was an exception for 60°, 75°, and 90° specimens, which are prone to fracture by an out-of-plane mode, where slight lateral support was retained to prevent global buckling. Additionally, lubricant was applied on the contact surfaces of specimen ends and loading blocks to minimize friction.

A GOM ARAMIS 4M 2D DIC system, equipped with a 4-megapixel CMOS camera and a 35 mm lens, was mounted in front of the test set-up to capture images during each test, allowing for the calculation of full-field deformation histories by means of the digital image correlation (DIC) technique. To facilitate DIC measurements, the specimen surfaces were initially coated with white paint and then treated with stochastic speckle patterns using black aerosol spray painting. Photography was recorded at a frequency of one frame per second during loading.

Based on the feature size of DIC patterns estimated from the zoom-in images, a facet size of 15 × 15 pixel${}^{2}$ was selected, with a facet step of 11 × 11 pixel${}^{2}$ to ensure sufficient spatial resolution. The central 3 × 3 mm${}^{2}$ rectangular region was viewed as a virtual strain gauge, within which discrete strain data were averaged to represent the total deformation of the specimen. The choice of the virtual gauge region size has a negligible impact on the results, indicating that the strain distribution in the central area of the off-axis specimen is essentially uniform. Detailed background noise analysis was not conducted, but a comparison was made with a traditional resistance strain rosette (0/45/90 degrees) attached to the back surface of the specimen. DIC results have shown good agreement with strain gauge measurements, while offering a broader measurable range.

## 3. Testing Results and Analysis

### 3.1. Experimental Observations

**Figure 3:**
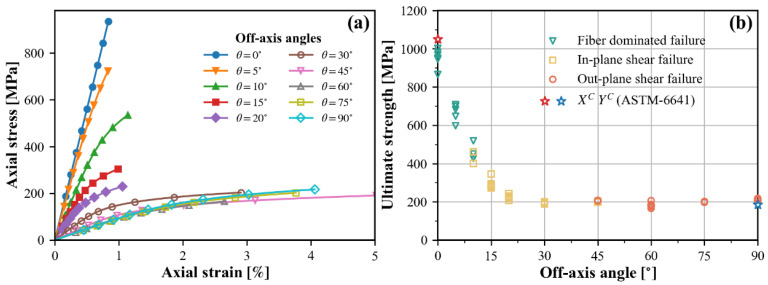
Experimental observations: (**a**) axial stress–strain curves, (**b**) failure strengths.

An overview of the direct test recordings can be seen in Figure 3. Axial stress is calculated by dividing the applied load by the cross-sectional area perpendicular to the loading axis. Thus, the ultimate strength can be defined as the absolute value of the axial stress at the peak load.

Figure 3a illustrates axial stress–strain curves. Due to the good repeatability of the curves in the same test group, one representative curve was selected for each on-axis/off-axis angle. All specimens undergo apparent nonlinearity prior to failure, except for the longitudinally compressed specimens. The curve for the 45° specimen group is not entirely displayed, and the strain can reach up to 10% to 12% eventually. The ultimate strengths of all specimens are plotted against off-axis angles in Figure 3b. The compressive strength decreases dramatically as the off-axis angle varies from 0° to 30°, and then remains relatively constant with only a slight increase. It is worth noting that the longitudinal and transverse compressive strengths of the small block specimens averaged 981.8 and 209.1, respectively, with relative errors of 3.7% and 3.0% when compared to the respective values obtained using the standardized method (Table 1). Hence, it can be confidently concluded that the basic strength properties determined in this test are reliable.

**Figure 4:**
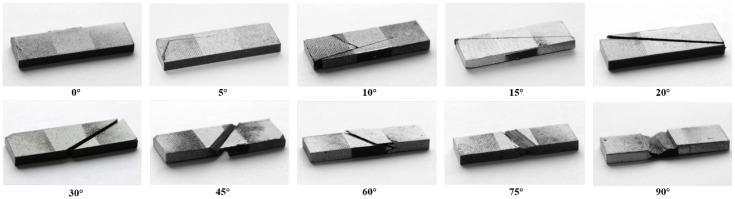
Failure modes of different specimens under off-axis compression.

**Figure 5:**
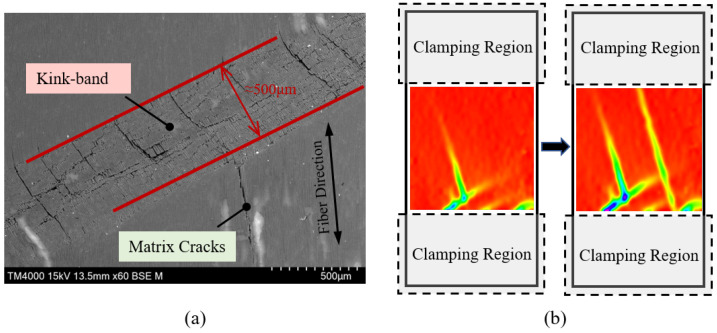
Observation of kink band formation: (**a**) electron micrograph of a 5° specimen; (**b**) DIC measurement of a 10° specimen.

The majority of specimens failed immediately after reaching their maximum load. Figure 4 illustrates typical failure modes for each group of specimens. The longitudinal compression specimens (namely 0°) failed due to fiber breakage, and no premature end collapse or splitting along the fiber direction was observed. This can be largely attributed to the manner in which compression was applied and the appropriate cross-sectional dimensions. Close inspection of the broken specimens revealed the presence of kink bands. For specimens with small off-axis angles, specifically 5° and some 10° specimens, failure was also dominated by fiber kinking. As shown in Figure 5a, localized shear deformation and fiber deflection occurred within a narrow band, with fiber breakage at both edges and in the interior. The width of the kink band can be roughly measured as 500 μm in the image. Although a small number of matrix cracks were observed in the electron micrographs, it can be confirmed that the formation of kink bands remains the primary factor contributing to ultimate failure.

As the off-axis angle increases, the occurrence of matrix cracking becomes more pronounced and eventually the dominant factor. In the 10° specimen group, apparent fiber kinking and matrix cracking were observed simultaneously in two specimens, while the other specimens were torn apart by a single matrix crack. Notably, the specimens that experienced fiber kinking had higher strength compared to those failed in a matrix-dominated mode. This suggests that friction on the contact surfaces was not entirely eliminated during the testing of these two specimens, and thus lateral constraint delayed the onset of matrix cracking. Figure 5b presents the axial compressive strain field of a 10° specimen captured by the DIC system approaching $t_{u}$, which denotes the time of ultimate failure. The visual depiction of deformation concentration in these images illustrates the evolution of kink bands and matrix cracks from their initiation to full development. However, the process unfolds so rapidly that these images by themselves do not conclusively demonstrate the correlation between the two failure mechanisms, i.e., whether fiber kinking is triggered by matrix cracking or vice versa.

When the off-axis angle is greater than 10°, matrix cracking parallel to the fiber orientation, primarily through the center of the specimen, becomes the dominant factor leading to failure. Two distinct failure modes can be further identified depending on the orientation of the fracture plane. For 15°, 20°, and 30° specimens, the fracture plane is nearly perpendicular to the specimen surface, where matrix failure is predominantly governed by in-plane shear. Conversely, for specimens with larger off-axis angles, such as 45°, 60°, 75°, and 90°, the fracture surface tends to be noticeably inclined, indicating a dominant influence of transverse compression.

**Figure 6:**
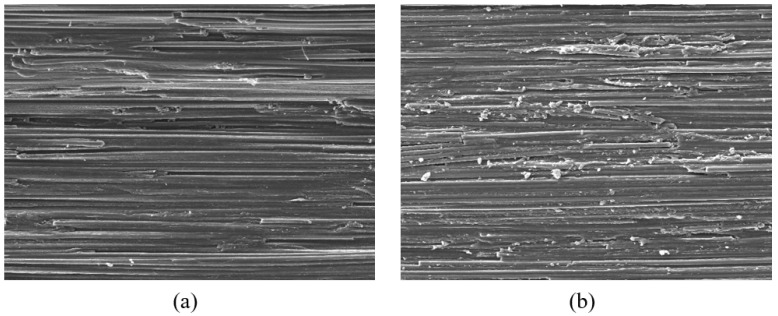
Electron micrographs of fracture surfaces: (**a**) in-plane shear mode in a 20° specimen; (**b**) transverse compression mode in a 60° specimen.

The electron microscope images of the fracture planes corresponding to in-plane shear mode and transverse compression mode are shown in Figure 6. It is observed that, apart from isolated fiber breakage due to surface abrasion, no signs of fiber failure were found. In contrast to the smoothness observed in the in-plane shear mode, the fracture plane appears rougher in the transverse compression mode. This can be explained from a microscopic perspective. Under transverse compression, micro-cracks in the matrix and fiber–matrix debonding predominantly advance and converge in a direction perpendicular to the fiber direction, resulting in uneven progression as they navigate through the gaps between fiber cross-sections.

In accordance with the brittle fracture hypothesis, it is not sufficient to describe the matrix-dominated failure behavior of unidirectional FRPs under off-axis compression using only the ultimate strength. This is because without information on the orientation of the fracture plane, it is not possible to determine the fracture resistance. Customarily, the inclination of the fracture plane is described by its angle with respect to the through-thickness direction, which can be readily measured from a side view of the fractured specimen. In the case of pure transverse compression (namely 90°), the fracture plane angle is typically deemed an intrinsic property closely associated with the material itself. In line with most findings in the literature [12,14], for unidirectional FRPs, this angle exceeds that of the plane with the maximum shear stress, suggesting a friction-like behavior of the fracture plane.

**Figure 7:**
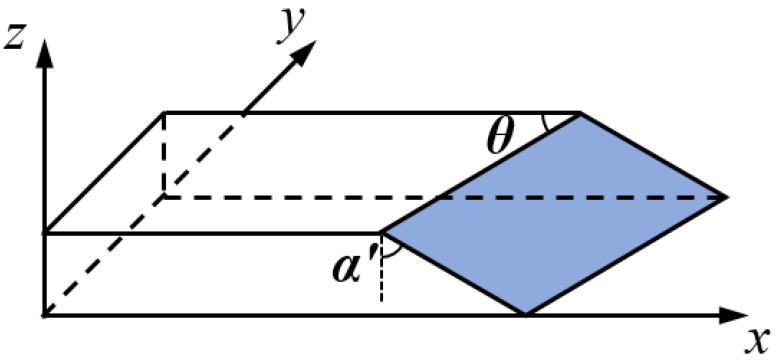
Determination of fracture plane angle for off-axis specimens.

It should be noted that, for off-axis specimens, the angle measured on the side surface does not accurately represent the fracture surface angle and a geometric transformation is required, as illustrated in Figure 7.

So, the fracture plane angle can be calculated as:

(1) $$\alpha_{\mathrm{fp}}=\frac{\pi }{2}-\arccos\left(n_{z}\cdot \left(n_{\theta }\times n_{\alpha }\right)\right)$$

where

(2) $$n_{z}=\left[0,0,1\right]\;\;\;\;\;n_{\theta }=\left[\cos\theta ,\sin\theta ,0\right]\;\;\;\;n_{\alpha^{\prime }}=\left[-\sin\alpha^{\prime },0,\cos\alpha^{\prime }\right]$$

It should be noted that intact fracture planes were not always found in broken specimens and secondary damage or irrecoverable deformation was evident in some of them. The measurements summarized in Table 2 may lack statistical significance and are for reference only.

### 3.2. Nonlinear Response

Customarily, the mechanical behavior of unidirectional fiber-reinforced composites is described in a material coordinate system with one axis coincident with the fiber direction and a second axis perpendicular to it in the plane. The transformation of stress and strain can be performed by:

(3) $$\sigma_{11}=\sigma_{x}\cos^{2}\theta \;\;\;\sigma_{22}=\sigma_{x}\sin^{2}\theta \;\;\;\;\tau_{21}=\sigma_{x}\sin\theta \cos\theta$$

and

(4) $$\begin{array}{c} \varepsilon_{11}=\varepsilon_{x}\cos^{2}\theta +\varepsilon_{y}\sin^{2}\theta +\gamma_{xy}\sin\theta \cos\theta \\ \varepsilon_{22}=\varepsilon_{x}\sin^{2}\theta +\varepsilon_{y}\cos^{2}\theta -\gamma_{xy}\sin\theta \cos\theta \\ \gamma_{21}=-2\left(\varepsilon_{x}-\varepsilon_{y}\right)\sin\theta \cos\theta +\gamma_{xy}\left(\cos^{2}\theta -\sin^{2}\theta \right) \end{array}$$

To facilitate the description of material nonlinear response, an elastic relation normally employed in the laminate analysis is assumed, where the modulus of elasticity $E_{2}$ and $G_{12}$ are replaced by the secant modulus $E_{2}^{s}$ and $G_{12}^{s}$. The strain components are calculated as follows:

(5) $$\left(\begin{array}{c} \epsilon_{11}\\ \epsilon_{22}\\ \gamma_{21} \end{array}\right)=\left[\begin{array}{ccc} 1/E_{1} & -v_{12}/E_{2}^{s} & 0\\ -v_{21}/E_{1} & 1/E_{2}^{s} & 0\\ 0 & 0 & 1/G_{21}^{s} \end{array}\right]\left(\begin{array}{c} \sigma_{11}\\ \sigma_{22}\\ \tau_{21} \end{array}\right)$$

where $\nu_{21}$ and $\nu_{12}$ are the larger and smaller Poisson’s ratio, which are linked to each other by the classical relation $\nu_{12}E_{1}=\nu_{21}E_{2}$.

It can be seen that the shear response is decoupled from the compressive (or tensile) response, In other words, $\sigma_{11}$ and $\sigma_{22}$ do not affect the shear strain $\gamma_{21}$, and $\tau_{21}$ does not affect the strains $\epsilon_{11}$ and $\epsilon_{22}$. It must be emphasized, however, that this decoupling is only reflected in the mathematical expression. Indeed, there is a clear interaction between the nonlinearities of transverse compression and in-plane shear, which is manifested in the secant modulus.

**Figure 8:**
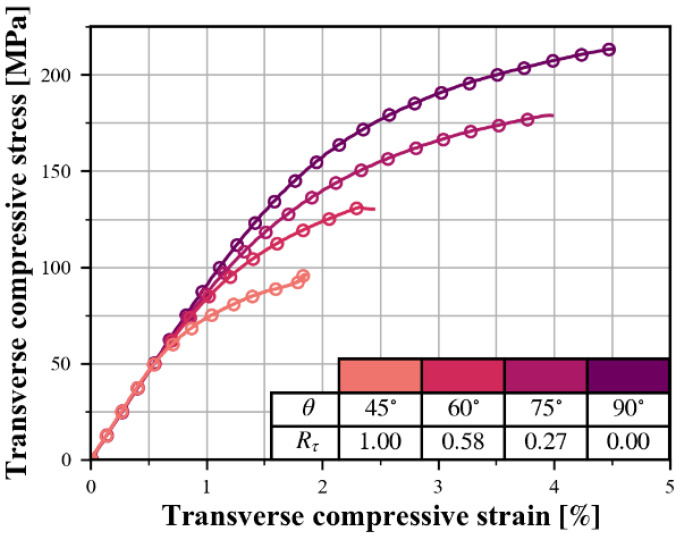
Typical transverse compressive curves combined with in-plane shear.

Figure 8 shows a typical transverse compression stress–strain response for various specimen groups, where each off-axis angle correspond to a specific stress ratio $R_{\tau }=\tau_{21}/\sigma_{22}$. It is worth noting that, in the off-axis loading condition, $\sigma_{11}$ and $\sigma_{22}$ act simultaneously and $\epsilon_{22}$ is a superposition of the transverse strain caused by both. To make it convenient to compare the curves for different off-axis angles, the horizontal axis represents the “combined strain” defined as

(6) $${\bar{\varepsilon }}_{22}=\frac{\varepsilon_{22}+\nu_{21}\varepsilon_{11}}{1-\nu_{21}\nu_{12}}$$

Compared to the stress–strain curve for uniaxial transverse compression, the curve obtained for off-axis compression is shifted downward, i.e., toward a lower transverse compressive stress $\left|\sigma_{22}\right|$, due to superimposed in-plane shear.

**Figure 9:**
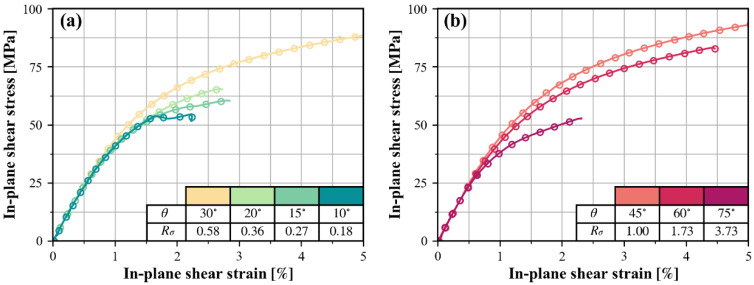
Typical in-plane shear curves combined with: (**a**) relative low transverse compression; (**b**) relative high transverse compression.

The situation is a bit more complicated for the in-plane shear response. As can be seen in Figure 9, the in-plane shear curve is shifted to higher $\left|\tau_{21}\right|$ in some cases, and to lower $\left|\tau_{21}\right|$ in others, depending on the stress ratio $R_{\sigma }=\sigma_{22}/\tau_{21}$.

As will be seen later, the coupling pattern between transverse compression and in-plane shear nonlinearities appears to have a certain connection with the matrix failure behavior under the same stress combination. A reasonable speculation is that the material nonlinearity is primarily induced by the matrix material and is associated with micro-damage within it. An in-depth study on non-linear material behavior is essential. However, this paper primarily focuses on failure mechanisms and strength prediction, while the constitutive behavior will be examined elsewhere.

### 3.3. Failure Loci in Two-Dimensional Diagrams

**Figure 10:**
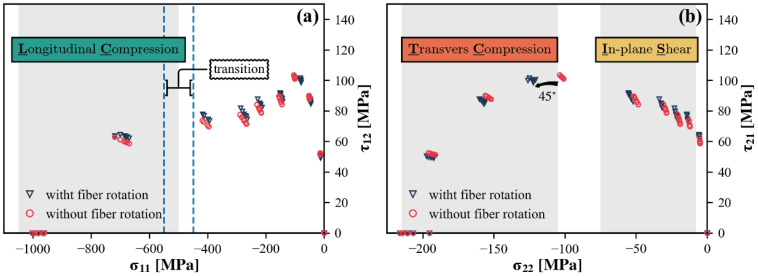
Failure loci presented in (**a**) $\sigma_{11}-\tau_{12}$ and (**b**) $\sigma_{22}-\tau_{12}$ stress planes.

For off-axis compression loading, the axial stress $\sigma_{x}$ is decomposed into three non-vanishing stress components: $\sigma_{11}$ for longitudinal compression, $\sigma_{22}$ for transverse compression, and $\tau_{12}$ for in-plane or axial shear, respectively. Through the coordinate transformation law, Figure 3b can be converted into two individual diagrams. One diagram pertains to the correlation between longitudinal compression and in-plane shear, while the other correlates transverse compression with in-plane shear. Bear in mind that one of the normal stress components has intentionally been omitted when illustrating the results of off-axis compression tests in each diagram. Hence, the contour outlined by failure loci does not strictly represent failure envelope in the two-dimensional stress space. For a proper understanding, Figure 10 annotates the valuable information conveyed by the two diagrams.

In the high longitudinal compression region of the $\sigma_{11}-\tau_{12}$ diagram, failure loci belong to small off-axis angle specimens that undergo fiber-dominated failure. In such cases, the transverse compression remains consistently at very low levels, allowing the results of the off-axis tests to be used to study longitudinal compression failure under the combined influence of $\sigma_{11}$ and $\tau_{12}$. Unfortunately, only the failure loci associated with the 5° specimens clearly fit into this region, making it impossible to accurately depict the variation trend. Nevertheless, this provides an important factual basis for the study of fiber compressive failure behavior, including the effect of in-plane shear. It can also be inferred that there is a transition zone where the failure mode shifts from fiber-dominated to matrix-dominated. As the fiber orientation varies, the dominant compression gradually changes from the longitudinal to transverse direction. Consequently, as the $\tau_{12}$-axis is approached, $\sigma_{22}$ is no longer negligible. The $\tau_{12}$ at matrix failure ascends to a peak surpassing the in-plane strength $S_{21}$ and then rapidly descends to the origin, signifying a pure transverse compression failure.

It is widely acknowledged that $\sigma_{11}$ contributes little to matrix-dominated failure. The off-axis test results displayed in the $\sigma_{22}-\tau_{21}$ diagram can be considered equivalent to biaxial test results, except for those failure loci associated with fiber-dominated failure. The interaction between $\sigma_{22}$ and $\tau_{12}$ is characterized by two obvious features, related to the transverse compression mode and the in-plane shear mode, respectively. At high transverse compression, a mutual weakening effect occurs, i.e., the transverse compressive stress $\sigma_{22}$ at failure is reduced by the applied shear stress $\tau_{12}$ and vice versa. Another trend is known as the shear strengthening phenomenon, where moderate transverse compression enhances the ability to withstand in-plane shear stresses.

Strictly speaking, the coordinate transformation angle should integrate the fiber rotation that occurs during the test process, which can be obtained from the deformation gradient tensor provided by the DIC calculation. This implies that $\sigma_{11}$ should always be in the current fiber direction. Such a co-rotational coordinate system is better suited to studying matrix-dominated failure, since matrix cracks always appear in planes parallel to the fibers. Yet, this does not raise any concern because fiber rotation is negligible for most specimens, except for those with off-axis angles close to 45°.

## 4. Analytical Studies

### 4.1. Brief Introduction on Physically Based Failure Theories

Faced with numerous efforts in this area, several researchers, including Li et al. [24] and Christensen [25,26], have taken a firm stance, asserting that failure theories lacking a strong foundation in physics and mathematics can never truly be considered robust. While this opinion may be somewhat radical, particularly from the standpoint of engineering applications, it underscores the critical role of physical soundness in the pursuit of a reliable failure description. In this context, several failure theories (often referred to as physically based) have outperformed other competitors and have gained recommendations from researchers and the design community. Notable among them are contributions by Hashin [15,16], Puck [18], Pinho [19,20], and various extensions [27,28,29].

In principle, the construction of a failure theory with physical considerations entails the dissection of different failure modes and the development of failure criteria based on the analysis of underlying mechanisms. The distinction between longitudinal and transverse failure in unidirectional FRPs has become a consensus, which can be traced back to Hashin, who laid the groundwork for failure criteria based on failure mechanisms. In 1973 [15], he instructively categorized unidirectional FRP failure into fiber-dominated and matrix-dominated, each of which was further subdivided into tensile and compressive modes in 1980 [16].

Traditionally, matrix failure has been formulated as a polynomial in the stress components (usually excluding $\sigma_{11}$). Many of these criteria are transplanted from the yield criteria of von Mises or Hill, which were originally developed for ductile materials. However, the matrix fails primarily in a plane parallel to the fiber direction and behaves in a brittle manner. A more convincing criterion for matrix failure can be developed by introducing the so-called ’action plane’, i.e., a plane in which fracture is likely to take place. Although the concept was initially mentioned in Hashin’s 1980 article, it was Puck [18] who pioneered putting it into practice and vividly described this matrix failure phenomenon as “inter-fiber fracture (IFF)”.

**Figure 11:**
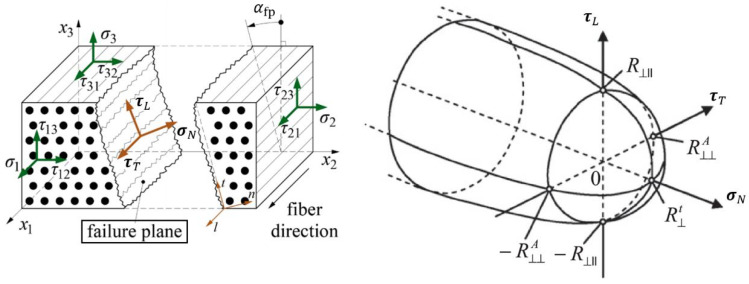
Visualization of the action plane and inter-fiber fracture condition.

Rooted in Mohr and Coulomb’s hypothesis, which was later adapted by Paul for transversely isotropic materials, the inter-fiber fracture condition is exclusively formulated with one normal and two shear traction components exerted on the action plane:

(7) $$F_{M}\left(\sigma_{N}\left(\alpha_{\mathrm{fp}}\right),\tau_{T}\left(\alpha_{\mathrm{fp}}\right),\tau_{L}\left(\alpha_{\mathrm{fp}}\right)\right)=1$$

where $\sigma_{N}$, $\tau_{T}$, and $\tau_{L}$ can be easily calculated using the transformation rules for a second-order tensor, as depicted schematically in Figure 11. For a generic stress state, the fracture plane angle $\alpha_{\mathrm{fp}}$ is unknown in advance, which calls for a search for the maximum value of $F_{M}$. Some efficient algorithms for carrying this out have been suggested [30,31].

Fiber kinking is a prevalent phenomenon in unidirectional fiber-reinforced composites, particularly those with a high fiber volume fraction, when exposed to intense longitudinal compression. There has long been an ongoing debate concerning the motivation behind the formation of kink bands. Microscopically, fibers within kink bands undergo a sudden change in direction, accompanied by localized deformation and damage. This abrupt loss of microstructural stability naturally evokes associations with fiber buckling under compression. However, mounting evidence [32,33] increasingly supports the contention that kink bands arise from a separate mechanism, distinct from the global micro-buckling mode proposed by Rosen [34].

Argon [35] was the first to establish the correlation between the longitudinal compressive strength $X_{C}$ and the in-plane shear strength $S_{21}$, by assuming that fiber kinking arises from local microstructural imperfections when matrix cracking takes place in the vicinity of the misaligned fibers. Afterwards, both analytical and numerical models based on the fiber-kinking theory (FKT) were developed to gain a deeper understanding of fiber compressive failure. Davila et al. [36] developed an FKT-based criterion in terms of homogenized in-plane stress components, which was subsequently extended to three-dimensional stress states by Pinho et al. [19].

**Figure 12:**
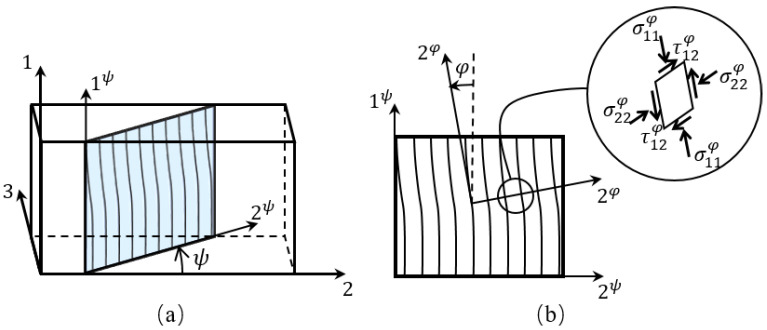
Illustration of fiber-kinking theory: (**a**) kinking plane; (**b**) misaligned coordinate.

Figure 12 illustrates the transformation procedure and relevant coordinate systems in the three-dimensional kinking model. To establish a fiber compression criterion in accordance with the FKT, the stress in a misaligned frame needs to be given by two successive transformations of coordinate systems, i.e.,

(8) $$\sigma^{\varphi }=T_{\varphi }\cdot T_{\psi }\cdot \sigma \cdot T_{\psi }^{T}\cdot T_{\varphi }^{T}$$

where $T_{\varphi }$ and $T_{\psi }$ are, respectively, the rotation tensor from 1-2-3 to $1^{\psi }-2^{\psi }-3^{\psi }$ and from $1^{\psi }-2^{\psi }-3^{\psi }$ to $1^{\varphi }-2^{\varphi }-3^{\varphi }$. Then, the onset of fiber kinking can be identified using a matrix failure criterion.

For a plane stress state under off-axis compression, the angle of the kinking plane, ψ, is assumed to be zero. The misalignment, φ, is the sum of an initial misalignment angle, assumed to be a material constant, and a rotation, $\gamma_{12}^{\varphi }$, induced by the applied shear stress in terms of the shear constitutive law expressed as $\gamma =f\left(\tau \right)$. So, $\gamma_{12}^{\varphi }$ can be determined by solving the following equation:

(9) $$f^{-1}\left(\gamma_{12}^{\varphi }\right)=-\frac{\left(\sigma_{11}^{\psi }-\sigma_{22}^{\psi }\right)}{2}\sin\left(2\left(\varphi_{0}+\gamma_{12}^{\varphi }\right)\right)+\left|\tau_{12}^{\psi }\right|\cos\left(2\left(\varphi_{0}+\gamma_{12}^{\varphi }\right)\right)$$

and the angle φ becomes:

(10) $$\varphi =\frac{\tau_{12}}{\left|\tau_{12}\right|}\left(\varphi_{0}+\gamma_{12}^{\varphi }\right)$$

To solve Equation (9) exactly, numerical methods such as the Newton–Raphson algorithm can be used (for details, see [20]). Assuming small angle approximations and a linear material response, the previous equation results in:

(11) $$G_{12}\gamma_{12}^{\varphi }\approx -\left(\sigma_{11}^{\psi }-\sigma_{22}^{\psi }\right)\left(\varphi_{0}+\gamma_{m}\right)+\left|\tau_{12}^{\psi }\right|$$

which can be solved in close form as:

(12) $$\gamma_{12}^{\varphi }=\frac{\varphi_{0}G_{12}+\left|\tau_{12}^{\psi }\right|}{G_{12}+\sigma_{11}^{\psi }-\sigma_{22}^{\psi }}-\varphi_{0}$$

Four representative failure theories are briefly described here: Hashin [16], Camanho [17], Puck [18], and Pinho [20]. The last one is more frequently referred to as the LaRC05 criteria, which amalgamate the wisdom of several predecessors, encompassing the concept of the action plane and fiber-kinking theory, as well as the in situ effect of strength. Distinct formulas for matrix failure and fiber failure are presented in Table 3 and Table 4, respectively. While some of them differ between tension and compression, the current discourse specifically centers on the compression scenario. To keep consistency, parameter symbols with analogous meanings across different theories have been unified.

The 1980 version of Hashin’s matrix criterion is adopted, which includes a linear term that enhances its flexibility in characterizing the failure envelope. Nevertheless, to circumvent the need for additional experimental measurements during parameter determination, it still incorporates certain simplifications, notably the controversial assumption of infinite equi-biaxial transverse compressive strength. Camanho provided an elegant and concise formulation for matrix failure by taking advantage of the material’s symmetry. In this formulation, $I_{1}$, $I_{2}$, and $I_{3}$ represent a set of irreducible stress invariants, while $\alpha_{1}$, $\alpha_{2}$, $\alpha_{3}^{c}$, and $\alpha_{32}^{c}$ are coefficients awaiting calibration through experimental results. Hashin’s criterion also falls under the category of invariant-based approaches but, owing to its widespread familiarity, it is usually expressed in terms of stress components and basic strength properties.

Both the Puck and LaRC05 theories employ the action-plane-related approach, defining matrix failure based on the IFF condition. In this context, traditional basic strengths are no longer applicable because they are not necessarily relevant to the fracture plane. Instead, fracture resistances, which represent the maximum sustainable normal or shear traction on the action plane when applied alone, should be incorporated into the fracture conditions. When compression is applied on a potential fracture plane, two resistances, $R_{⊥\|}^{A}$ and $R_{⊥⊥}^{A}$, play a role against fractures caused by $\tau_{L}$ and $\tau_{T}$, respectively. This is because a negative $\sigma_{N}$ only impedes fractures by increasing shear fracture resistances (or, equivalently, by reducing the effective shear traction [20]). To account for the increase in shear fracture resistances, two additional parameters $p_{⊥\|}^{\left(-\right)}$ and $p_{⊥⊥}^{\left(-\right)}$ are involved, which can be physically interpreted as the friction coefficients of the fracture plane in the sense of the Mohr–Coulomb law, when a linear effect of $\sigma_{N}$ is assumed.

For better consistency with experimental data, Puck and his colleagues introduced several refinements to the IFF condition. The version utilized in this section is the most widely accepted, distinguished by its notable feature that the fracture body displays analogous parabolic contours on any longitudinal sections rotated about the $\sigma_{N}$-axis. The matrix-dominated criterion in LaRC05 draws inspiration from Puck’s theory while broadening its capabilities to include the influence of adjacent plies or external boundaries. However, for sufficiently thick unidirectional laminates, the so-called in situ effect is absent, indicating that the matrix compression criterion in LaRC05 essentially mirrors the original Puck criterion.

With regard to fiber compression failure, the maximum stress criterion continues to be extensively employed, even though Hashin and Puck have independently elucidated the underlying mechanisms in their theories. In contrast, both Camanho and Pinho embrace the FKT-based criterion, with the only distinction being their individual methodologies for detecting matrix failure in the misaligned coordinate system.

According to Pinho’s explanation [37], localized damage to the supporting matrix only develops into a kink band when the compression in the longitudinal direction is extremely high; otherwise, it leads to splitting, which is essentially a matrix-dominated failure (or inter-fiber fracture). The transition between kinking and splitting is hard to identify, as they share the same expression and there is no specific change in the trend of the failure envelope. Experimental research by Jelf and Fleck [6] found that specimens only fail in the form of splitting when the longitudinal compression is low and in-plane shear is high. Accordingly, the transition between kinking and splitting can be roughly predicted based on the compressive stress in the fiber direction at the point of failure. In detail, fiber kinking occurs when $\left|\sigma_{11}\right|\geq X_{C}^{trans}$, while fiber–matrix splitting occurs when $\left|\sigma_{11}\right|<X_{C}^{trans}$. This issue was not explicitly mentioned by Camanho, but it can be guessed that the same treatment was adopted.

### 4.2. Input Parameters Related to Failure Criteria

During the application and evaluation of different failure theories, inaccurately determined or misinterpreted parameters not only introduce errors but, more critically, lead to illogical conclusions. The conventional methods for determining strength parameters in the four failure theories are described below.

The parameters required in Hashin’s matrix failure criterion have been measured and listed in Table 1, with the only exception being for the transverse shear strength $S_{23}$. The determination of $S_{23}$ poses some difficulties but, fortunately, there seems to be an inherent relationship between $Y_{T}$ and $S_{23}$. According to [27], as long as $Y_{C}/Y_{T}>3.45$, it is reasonable to approximate that:

(13) $$S_{23}=Y_{T}$$

For an invariant-based formulation of matrix failure in Camanho theory, it is easy to see that:

(14) $$\alpha_{1}=\frac{1}{S_{23}^{2}}\;\;\;\;\alpha_{2}=\frac{1}{S_{21}^{2}}$$

However, relying on uniaxial compression results alone is insufficient for determining the remaining two unknown parameters. It is recommended to introduce hydrostatic or equi-biaxial transverse compressive strength, denoted as $Y_{BC}$, so that:

(15) $$\alpha_{3}^{c}=\frac{1}{2Y_{BC}}-2\alpha_{32}^{c}Y_{BC}\;\;\;\;\alpha_{32}^{c}=\frac{1-Y_{C}/2Y_{BC}-\alpha_{1}Y_{C}^{2}/4}{Y_{C}^{2}-2Y_{BC}Y_{C}}$$

A total of four parameters are required for IFF conditions in either Puck or LaRC05 (an additional three parameters when the tensile mode is considered). It is acknowledged that a pure longitudinal shear loading (e.g., $\tau_{21}$ or $\tau_{31}$) always causes fracture in its action plane; thus, the corresponding fracture resistance $R_{⊥\|}^{A}$ is equal to $S_{21}$. The situation is quite different for $R_{⊥⊥}^{A}$, as pure transverse shear stressing (e.g., $\tau_{23}$) typically leads to tensile fracture in the plane of maximum principal stress. In view of this, $R_{⊥⊥}^{A}$ is measured indirectly through a transverse compression test. The critical condition is reached when the Mohr circle corresponding to transverse compressive loading touches the $\sigma_{N}-\tau_{T}$ fracture curve. $p_{⊥⊥}^{\left(-\right)}$ can be determined simultaneously since two curves are tangent at this point. Therefore, the values of $R_{⊥⊥}^{A}$ and $p_{⊥⊥}^{\left(-\right)}$ can be calculated from the compressive strength $Y_{C}$ and the measured fracture angle $\alpha_{\mathrm{fp}}$.

For Puck criterion:

(16) $$p_{⊥⊥}^{\left(-\right)}=\frac{1}{2\cos^{2}\alpha_{\mathrm{fp}}}-1\;\;\;\;R_{⊥⊥}^{A}=\frac{Y_{C}}{2\left(1+p_{⊥⊥}^{\left(-\right)}\right)}\;\;\;\;R_{⊥\|}^{A}=S_{21}$$

For LarC05 criterion:

(17) $$p_{⊥⊥}^{\left(-\right)}=-\frac{1}{\tan\left(2\alpha_{\mathrm{fp}}\right)}\;\;\;\;R_{⊥⊥}^{A}=\frac{Y_{C}}{2\tan\alpha_{\mathrm{fp}}}\;\;\;\;R_{⊥\|}^{A}=S_{21}$$

In addition, a simplification of the coupling between the inclination parameters $p_{⊥⊥}^{\left(-\right)}$ and $p_{⊥\|}^{\left(-\right)}$ is introduced as

(18) $$\frac{p_{⊥⊥}^{\left(-\right)}}{R_{⊥⊥}^{A}}=\frac{p_{⊥\|}^{\left(-\right)}}{R_{⊥\|}^{A}}=\left(\frac{p}{R}\right)=const.$$

With regard to fiber compression failure, Hashin and Puck utilize the maximum stress criterion, relying directly on the uniaxial strength property $X_{C}$. In the FKT-based criterion embraced by Camanho and Pinho, the initial fiber misalignment angle $\varphi_{0}$ plays a pivotal role and is usually calculated by considering the critical condition that the material fails under pure longitudinal compression, i.e., $\sigma_{11}=X_{C}$. Assuming that $\varphi_{C}$ is the total misalignment angle at the moment of failure, the stresses in the misalignment coordinate system are:

(19) $$\sigma_{11}^{\varphi }=-X_{C}\cos^{2}\varphi_{C}\;\;\;\;\sigma_{22}^{\varphi }=-X_{C}\sin^{2}\varphi_{C}\;\;\;\;\tau_{12}^{\varphi }=X_{C}\sin\varphi_{C}\cos\varphi_{C}$$

By substituting them into the matrix failure criteria of Camanho and LaRC05, respectively, the values of $\varphi_{C}$ can be determined. The initial misalignment angle is then calculated by reversing the deformed rotation. For a material that exhibits linear response in shear, $\varphi_{0}$ can be calculated as follows:

(20) $$\varphi_{0}=\varphi_{C}-\frac{X_{C}\sin2\varphi_{C}}{2G_{12}}\approx \varphi_{C}\left(1-\frac{X_{C}}{G_{12}}\right)$$

Curiously, there is a scarcity of experimental studies concerning the validation and measurement of such fiber imperfections.

All the parameters in these failure criteria are determined a priori, summarized in Table 5.

### 4.3. Comparison with Experimental Results

**Figure 13:**
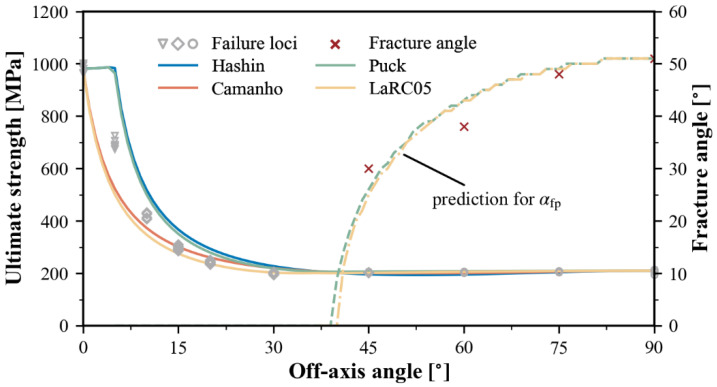
Predictions of off-axis compression strength.

The predicted ultimate strength as a function of the off-axis angle according to various failure theories is displayed in Figure 13, alongside the experimental results. All predicted curves show a similar trend to the experimental results, but there are some differences in the range of small off-axis angles. The Hashin and Puck theories overestimate the compressive strength in the range of 0–30°, while the LaRC05 theory shows an underestimation in the same range. When the loading direction starts to deviate from 0°, the predicted curve of the Camanho theory initially coincides with that of the LaRC05 theory, but it agrees slightly better with the experimental results at 15° and 20°.

Figure 13 also presents the variation in fracture angles predicted by two theories, Puck and LaRC05, respectively. For small off-axis angles, the inclination of the fracture plane remains unnoticeable. However, as the off-axis angle increases, the transverse compression begins to dominate, resulting in a rapid increase in the fracture angle, eventually reaching approximately 51° degrees under pure transverse compression. It can be concluded that the action-plane-related matrix criterion can accurately identify the transition between the two matrix failure modes of transverse compression and in-plane shear.

The predictive capabilities of different theories can be compared more intuitively in two-dimensional diagrams depicting $\sigma_{11}-\tau_{12}$ and $\sigma_{22}-\tau_{21}$. The predicted curves generated by each theory encompass scenarios of both off-axis and ideal biaxial compressive loading, delineated by solid and dashed lines, respectively. The latter actually represents the theoretical failure envelope in the two-dimensional stress space, and is presented for comparative analysis of the off-axis test with the biaxial test from a theoretical point of view.

**Figure 14:**
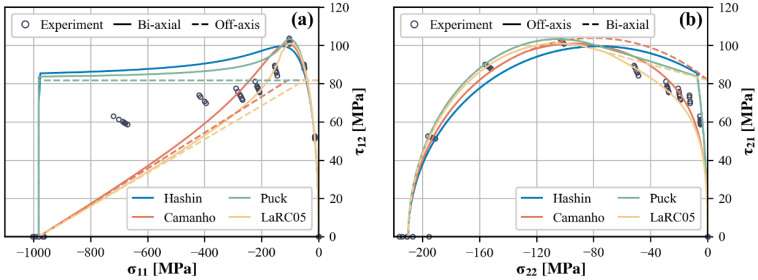
Predictions of off-axis compression tests from different failure theories in both (**a**) $\sigma_{11}-\tau_{12}$ and (**b**) $\sigma_{22}-\tau_{21}$ stress planes.

Figure 14a illustrates the different predictions for the stress combination $\sigma_{11}-\tau_{12}$. In the high longitudinal compression region, the predictions exhibit two distinct patterns, reflecting different considerations of fiber failure. For the Hashin and Puck theories, the longitudinal compressive strength is insensitive to shear stress, so their predicted curves take on a rectangular or nearly rectangular shape. In contrast, the Camanho and LaRC05 theories predict an approximately linear relationship with the assumption that the presence of in-plane shear stress promotes the formation of kink bands. Comparison with experimental results indicates that neither the maximum stress nor the FKT-based criteria agree well with the experimental data. Specifically, the failure loci measured by the off-axis compression tests fall between the linear and rectangular predictions.

It has been stated that the off-axis testing results depicted do not fully represent the failure envelope in this two-dimensional stress plane, as evidenced by the noticeable disparity between the predicted curves for off-axis and biaxial loading in the region of low longitudinal compression. Since all four failure theories invariably assume that matrix failure is independent of $\sigma_{11}$, there is a common upper limit at $\tau_{12}=S_{21}$ among the predicted envelopes, which represents an in-plane shear failure resulting from $\tau_{12}$ alone. When dealing with off-axis loading, the effect of the third stress component $\sigma_{22}$ beyond this diagram is addressed through different matrix failure criteria.

A more comprehensive illustration and comparison of the predictions for matrix-dominated failure is attainable in the $\sigma_{22}-\tau_{21}$ diagram, as shown in Figure 14b. Under relatively high transverse compression, the predictions of Comanho, Puck, and LaRC05 closely match the experimental results, whereas the prediction of Hashin shows a slight underestimation. In the region of high in-plane shear, while all of the predicted curves capture the shear strengthening effect to some extent, they show greater differences from each other. Both the Hashin and Puck criteria overestimate $\tau_{21}$ at failure when superimposed on a relatively low compressive $\sigma_{22}$, but Puck’s prediction shows a better fit to the variation trend. At first glance, the curves predicted by Comanho and LaRC05 appears to agree well with the experimental results, especially with the latter effectively reproducing the near-linear enhancement of $\tau_{21}$ by compressive $\sigma_{22}$. However, it should be noted that their predictions in this region are not determined by the matrix failure criterion but rather controlled by the FKT-based criterion for fiber failure, which appears to be inconsistent with the observed failure modes. According to the theoretical introduction, this problem can be eliminated by choosing a suitable $X_{C}^{trans}$. Nevertheless, the predicted results of Camanho and LaRC05 are still questionable as they are at odds with the previous discussion of the experimental results, i.e., the failure loci projected onto this stress plane roughly coincides with the two-dimensional failure envelope. There may be some misunderstanding about the initial misalignment angle in the FKT-based criterion.

The predictions for ideal biaxial loading indirectly support this suspicion, as all four predicted envelopes are non-conservative relative to the experimental results. Graphically, these envelopes converge at the same point on the $\tau_{21}$-axis that corresponds to failure under pure in-plane shear. Consequently, there is further speculation that the unsatisfactory predictions are not solely attributed to the criteria themselves; an accurate determination of the in-plane shear strength $S_{12}$ carries a parallel significance.

### 4.4. Recalibration with Off-Axis Test Results

In case $Y_{BC}$ is not accessible, Camanho et al. [17] alternatively suggest resorting to off-axis testing. However, they do not explicitly require whether the off-axis results should be from tensile or compressive tests, nor do they specify off-axis angles. It is only emphasized that the critical stress state must be taken from a matrix-dominated failure. Based on a combination of stress components ($\sigma_{22u}$, $\tau_{21u}$), the parameters are determined as follows:

(21) $$\begin{array}{c} \alpha_{3}^{c}=1/Y_{C}-\alpha_{1}Y_{C}/4-\alpha_{32}^{c}Y_{C}\\ \alpha_{32}^{c}=\frac{1-\left(1/Y_{C}-\alpha_{1}Y_{C}/4\right)\sigma_{22u}-\alpha_{1}^{2}\sigma_{22u}/4-\alpha_{2}\tau_{21u}^{2}}{\sigma_{22u}^{2}-Y_{C}\sigma_{22u}} \end{array}$$

**Figure 15:**
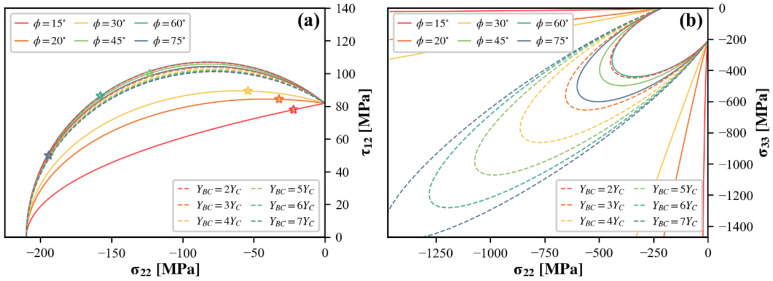
Guidance for selecting off-axis test data to calibrate the Camanho criterion (star symbols represent the test data): (**a**) $\sigma_{22}-\tau_{12}$ and (**b**) $\sigma_{22}-\sigma_{33}$ stress planes.

Predicted curves calibrated with different off-axis test data are presented in Figure 15, together with those generated for a variety of assumed $Y_{BC}$ values. It can be observed that only the test results for large off-axis angles can be relied upon. In the low compression region, the elliptical curves dictated by the formulation of the Camanho criterion do not truthfully fit the experimental failure envelope. Thus, if the curve is forced to pass through a particular failure locus within this region, the overall prediction will be severely biased. It should also be noted that calibration results derived from off-axis tests are more sensitive to experimental errors, as evidenced by the predicted envelopes in the $\sigma_{22}-\sigma_{33}$ stress plane. Since having a closed failure envelope in this stress plane is a major advantage of the Camanho theory, it is essential to verify that the estimates of $Y_{BC}$ are within acceptable limits after being calibrated by off-axis testing results.

**Figure 16:**
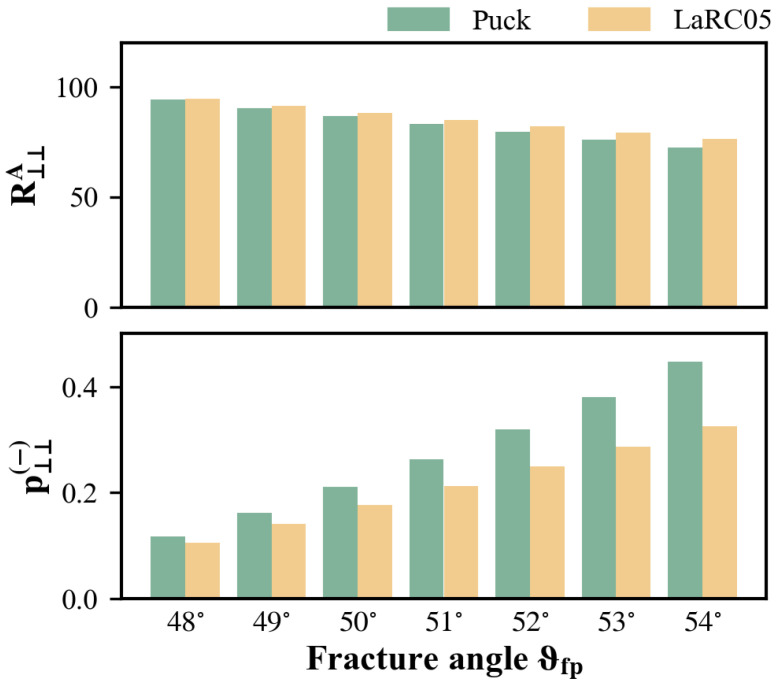
Influence of fracture angle measurement on determination of $R_{⊥⊥}^{A}$ and $p_{⊥⊥}^{\left(-\right)}$.

As mentioned earlier, there is a need for an accurate method to determine the in-plane shear strength $S_{21}$. Tsai et al. [13] previously proposed that the pure in-plane shear strength could be extrapolated from off-axis test results. However, at that time, their focus was solely on the shear strength values, without clarifying the exact form and meaning of the extrapolation function. In addition, it is noticed that the derivation process for the parameters involved in action-plane-related IFF conditions relies on the measurement of the fracture angle, which tends to be quite scattered. As depicted in Figure 16, $R_{⊥⊥}^{A}$ and $p_{⊥⊥}^{\left(-\right)}$ are sensitive even to marginal changes in the fracture angle, and this sensitivity is also transferred to $p_{⊥\|}^{\left(-\right)}$ via Equation (18). Consequently, it is sometimes more convenient to directly select a typical value for $p_{⊥\|}^{\left(-\right)}$ [38]. It must be pointed out that the suggested range for $p_{⊥\|}^{\left(-\right)}$ and the assumptions about parameter coupling are derived empirically in the absence of experimental data. Utilizing them without further validation carries a certain risk; thus, more dependable methods are required.

**Figure 17:**
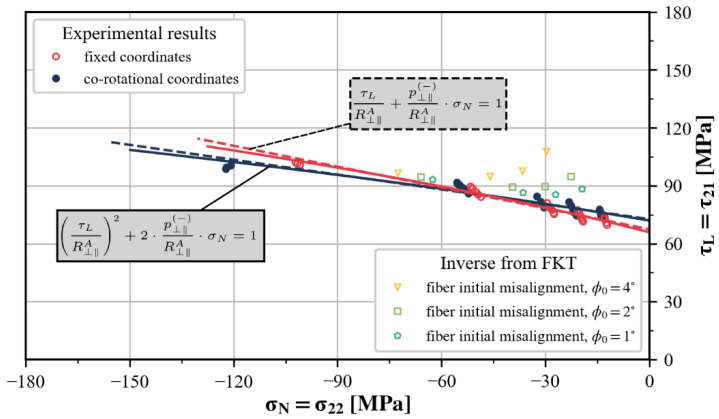
Parameter determination of $R_{⊥\|}^{A}$ (or $S_{21}$) and $p_{⊥⊥}^{\left(-\right)}$.

Following Puck’s suggestion, the parameter $p_{⊥\|}^{\left(-\right)}$ can be derived from the experimentally established $\sigma_{22}-\tau_{21}$ envelope since the fracture plane angle is zero in this segment of the failure envelope, where $\sigma_{N}=\sigma_{22}$ and $\tau_{L}=\tau_{21}$. Combining these pieces of information, the values of $R_{⊥\|}^{A}$ (or $S_{21}$) and $p_{⊥⊥}^{\left(-\right)}$ in the matrix failure criteria of Puck and LaRC05 can be determined by fitting the outcomes of off-axis compression tests, as shown in Figure 17. It can be seen that whether or not fiber rotation is taken into account has a certain but very limited impact on the parameter determination. For the sake of theoretical self-consistency, experimental results presented in co-rotational coordinates are preferred. Accordingly, fiber rotation must also be considered during failure prediction, which is roughly carried out in this paper by solving Equation (10) with $\varphi_{0}=0$.

In order to avoid uncertainties in the measurement of the fracture angle, the off-axis test results were also used to calibrate the predictions of the Puck and LarC05 codes in the high transverse compression range within the stress plane $\sigma_{22}-\tau_{21}$. Whether there exists an analytical solution of $R_{⊥⊥}^{A}$ and $p_{⊥⊥}^{\left(-\right)}$ by explicitly eliminating $\alpha_{\mathrm{fp}}$ depends on the specific form of the IFF condition. However, in most cases, it can be solved numerically.

**Figure 18:**
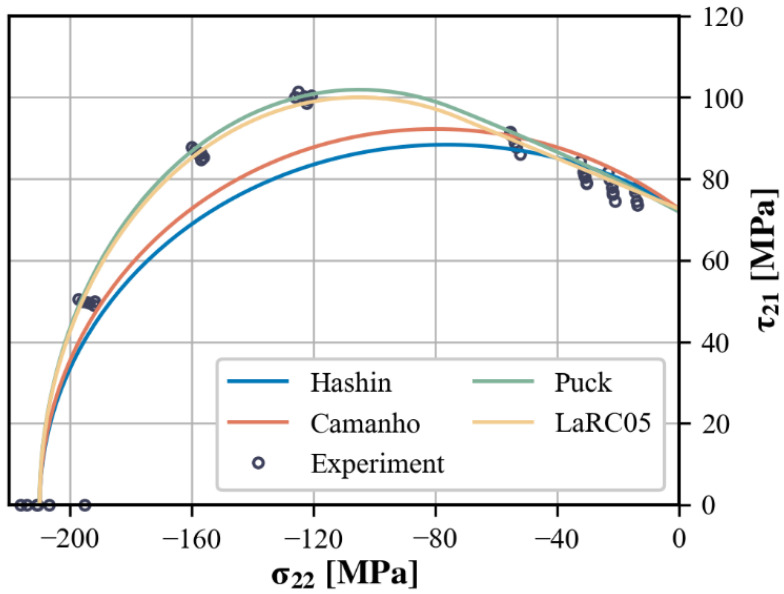
Predictions of the $\sigma_{22}-\tau_{21}$ failure envelope after recalibration with off-axis test results.

The validity of matrix failures predicted by FKT-based criteria has not been further explored until this point in the paper. As a result, only the matrix criteria from each failure theory are employed here to predict the failure envelope in the $\sigma_{22}-\tau_{21}$ stress plane. The results are shown in Figure 18.

Because of the limitations of their mathematical forms, the matrix failure predictions by Hashin and Camanho struggle in simultaneously conforming to the failure envelope trends for both high and low transverse compression. When the in-plane shear strength is tuned down, although there is a slight improvement in the predictions near the $\tau_{21}$-axis, the weakening effect of high compressive $\sigma_{22}$ on $\tau_{21}$ is irrationally amplified. Conversely, the action-plane-related IFF criteria, represented by Puck and LaRC05, show superior performance. Not only do they better approximate the experimental results, but they also identify the shift in failure modes.

### 4.5. Further Discussion on Fiber Compressive Failure

In describing fiber failure under the combined longitudinal compression and in-plane shear, neither the maximum stress criterion nor the FKT criterion effectively takes into account the influence of shear stress. The former, as one of the most traditional and straightforward failure criteria, bluntly disregards the influence of $\tau_{12}$. On the contrary, the latter seems to exaggerate the weakening effect of $\tau_{12}$ on the longitudinal compressive strength, probably stemming from an overestimation of microstructural imperfections.

In the current FKT model, fiber-dominated failure is initiated exclusively by matrix failure in the region of misaligned fibers. Therefore, there must be an initial misalignment angle to ensure the formation of a kink band under pure longitudinal compression conditions. Taking the material studied in this paper as an example, its initial misalignment angle, as determined from the longitudinal compressive strength, is approximately 4°. This value is slightly above the suggested range [39], and well above the standard deviation of fiber misalignment reported in [9], i.e., the standard deviation in the fiber direction is only about 0.5° for both unidirectional and quasi-isotropic laminates.

Figure 17 also validates a question raised above as to whether the initial fiber misalignment angle in the FKT model is real and continues to have an effect even though longitudinal compression is not a major factor. Assuming initial misalignment angles of 1°, 2°, and 4°, and considering their role in specimen failure at off-axis angles ranging from 10° to 30°, it is not difficult to invert the critical stresses for matrix failure in the deflection coordinates of the FKT model. These outcomes and their variation patterns contradict most of the existing experimental and theoretical findings. Therefore, it can be concluded that the determination and interpretation of the initial fiber deflection angle in the FKT-based criterion needs to be re-examined.

From a microscopic perspective, fiber kinking can be viewed as a chain reaction resulting from a microstructural disruption that is multifactorial. Recent experimental evidence suggests that kink bands originate not only in regions of severe fiber misalignment but also in regions where fiber rotation has been induced by other failure mechanisms. Gutkin et al. [40] conducted an experimental study on the compressive failure of single-edge notched carbon/epoxy specimens under longitudinal compression, with particular insight into the sequence of events leading to failure at the microscale. Failure initiates in the 0°-ply as a 45° crack near the notch and subsequently evolves into a kink band due to fiber rotation at the crack front. In situ and post mortem fractographic analysis of the failure process, coupled with numerical discussions presented in a separate paper [41], confirms the existence of shear-driven fiber compression failure in addition to the well-known kinking/splitting.

**Figure 19:**
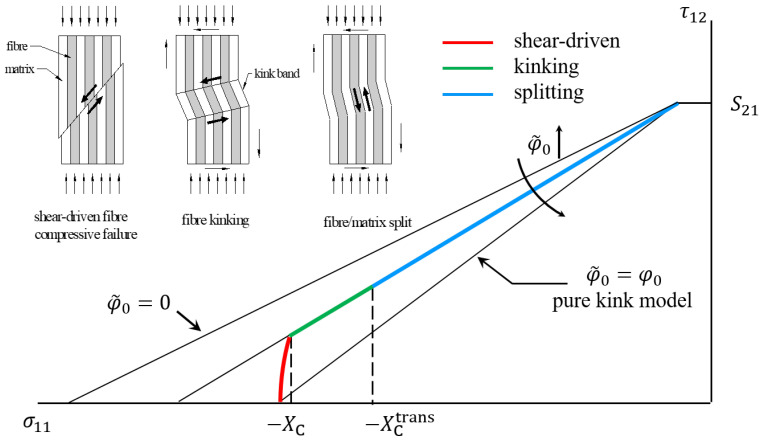
$\sigma_{11}-\tau_{12}$ failure envelope construction.

Informed by these facts, it is rational to speculate that the $\sigma_{11}-\tau_{12}$ failure envelope should be composed of two segments, as schematically drawn in Figure 19: the first runs nearly parallel to the $\tau_{12}$-axis in the high compression region and the second approximates a straight line with a positive slope. The location of the segment for shear-driven failure along the $\sigma_{11}$-axis is determined based on the longitudinal strength $X_{C}$. As a rule of thumb, a modest influence of the shear stress should also be considered. In practice, however, the experimental data needed to calibrate the parameters of interest are often lacking, so the maximum stress criterion is still adopted as an approximation. On the other hand, the slope of the segment associated with kinking/splitting depends on the initial fiber misalignment angle, which is now an independent parameter denoted as ${\tilde{\varphi }}_{0}$ for distinction. When $X_{C}$ is very high or ${\tilde{\varphi }}_{0}$ is very large, the first segment vanishes completely, thus degenerating into the classic FKT-based criterion.

**Figure 20:**
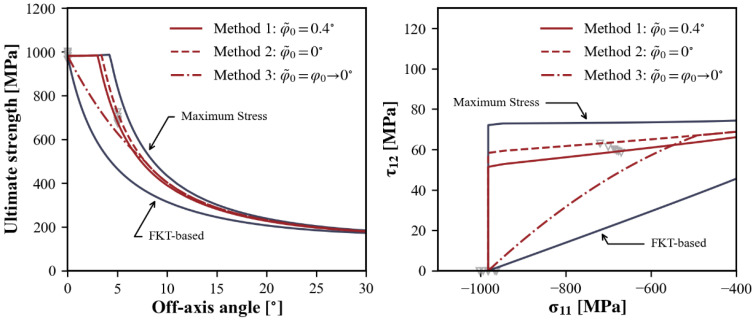
Modifications to fiber compressive failure criteria.

Figure 20 illustrates some simple modifications to the FKT-based criterion. ${\tilde{\varphi }}_{0}$ is determined by iteratively computing the $\sigma_{11}-\tau_{21}$ failure envelope to optimize its alignment with experimental results in the range of $\sigma_{11}<-X_{C}^{trans}$ (Method 1). Notably, the value of ${\tilde{\varphi }}_{0}$ is significantly smaller than the $\varphi_{0}$ of the pure FKT model. This implies that the microstructural imperfections do not appear to be as severe as originally anticipated and a prediction with ${\tilde{\varphi }}_{0}=0$ is perfectly acceptable (Method 2). Henceforth, the criteria for kinking/splitting and matrix cracking can be harmonized, provided that the matrix failure criterion is checked in a co-rotational coordinate system Another possible modification is to assume that the influence of initial fiber imperfections gradually diminishes as the longitudinal compression decreases. Thus, ${\tilde{\varphi }}_{0}$ is defined as a function of $\sigma_{11}$, which decreases from $\varphi_{0}$ at ${-X}_{C}$ to 0° at $-X_{C}^{trans}$ (Method 3). It is seen that, with these improvements, the prediction of pure longitudinal compression remains unchanged, but there is a noticeable improvement in the case of small off-axis angles or combined in-plane shear.

Overall, there are still challenges in accurately predicting fiber compressive failure under combined or off-axis loads. Some of the underlying failure mechanisms may not yet be fully comprehended. The proposed modifications to FKT-based criterion demonstrate improved agreement with experimental results, but further validation regarding the reasonableness and robustness of these predictions is needed.

## 5. Conclusions

This paper details a study on the compressive failure of unidirectional CCF300/BA9916-II composites, combining experimental and analytical insights. The study explores the nonlinear response and varied failure modes using block-shaped and end-loaded specimens under on-axis and off-axis compression. It delves into the impact of fiber orientation on failure strength and mode, along with the interaction between longitudinal/transverse compression and in-plane shear, to understand underlying mechanisms. Additionally, four failure theories (Hashin, Comanho, Puck, and LaRC05) are compared and assessed for their predictive capabilities. Recalibration and minor refinement were also implemented. Key conclusions include:

- Off-axis compression testing is an effective method for analyzing the failure behavior of unidirectional fiber-reinforced polymers under combined or off-axis stress. Despite limitations in load application, adjusting fiber orientations helps to manipulate stress ratios, covering typical compressive failure modes and characteristics. Simplicity in specimen geometry and testing, along with minimal equipment needs, make this method feasible, especially for block-shaped specimens under end-loaded compression.
- Pure in-plane shear strength and longitudinal/transverse compressive strengths, aligned with standard test methods, can be derived from off-axis testing. This unified testing setup can acquire all necessary material parameters for characterizing unidirectional FRPs under combined compression–shear loading, which is appealing for engineering practice.
- Action-plane-related criteria demonstrate systematic superiority in both the qualitative and quantitative description of matrix-dominated failures, predicting both failure stresses and fracture plane orientation. For fiber-dominated failures, maximum stress and FKT-based criteria show limitations, although the latter explains kink band formation well.
- The predictive performance of these criteria is significantly influenced by model parameters, often requiring empirical determination or recalibration. A refined fiber compression failure criterion, recalibrated with off-axis compression test results, shows improved alignment with experimental data.

## Figures and Tables

**Table 1:** Moduli and strength values for the CCF300/BA9916-II carbon/epoxy system.

Property	Description	Method	Value ^1^
$Y_{T}$	Transverse tensile strength	ASTM D3039 [21]	55
$E_{2}$	Transverse compressive modulus	ASTM D6641 [22]	9.2
$Y_{C}$	Transverse compressive strength	ASTM D6641	203
$E_{1}$	longitudinal compressive modulus	ASTM D6641	118.7
$X_{C}$	longitudinal compressive strength	ASTM D6641	1020
$G_{21}$	In-plane shear modulus	ASTM D7078 [23]	4.9
$S_{21}$	In-plane shear strength	ASTM D7078	82

^1^ Strength in MPa and modulus in GPa. All strength values are given in positive.

**Table 2:** Measurements of the fracture angle [°].

Off-Axis Angle	Min.	Max.	Avg.
45°	22	35	30
60°	32	48	38
75°	44	50	48
90°	50	54	51

**Table 3:** Failure criteria for matrix-dominated compressive failure.

Theory	Description
Hashin	$\left[{\left(\frac{Y_{C}}{2S_{23}}\right)}^{2}-1\right]\frac{\sigma_{22}+\sigma_{33}}{Y_{C}}+\frac{\tau_{21}^{2}+\tau_{31}^{2}}{S_{21}^{2}}+\frac{{\left(\sigma_{22}+\sigma_{33}\right)}^{2}}{4S_{23}^{2}}+\frac{\tau_{23}^{2}-\sigma_{22}\sigma_{33}}{S_{23}^{2}}=1$
Camanho	$\alpha_{1}I_{1}+\alpha_{2}I_{2}+\alpha_{3}^{c}I_{3}+\alpha_{32}^{c}I_{3}^{2}=1$
	$I_{1}=\frac{1}{4}\sigma_{22}^{2}-\frac{1}{2}\sigma_{22}\sigma_{33}+\frac{1}{4}\sigma_{33}^{2}+\tau_{23}^{2}\;\;\;I_{2}=\tau_{21}^{2}+\tau_{31}^{2}\;\;\;I_{3}=\sigma_{22}+\sigma_{33}$
Puck	${\left(\frac{\tau_{⊥\psi }}{R_{⊥\psi }^{A}}\right)}^{2}+2\cdot \frac{p_{⊥\psi }^{\left(-\right)}}{R_{⊥\psi }^{A}}\cdot \sigma_{N}=1$
	${\left(\frac{\tau_{⊥\psi }}{R_{⊥\psi }^{A}}\right)}^{2}={\left(\frac{\tau_{T}}{R_{⊥⊥}^{A}}\right)}^{2}+{\left(\frac{\tau_{L}}{R_{⊥\|}^{A}}\right)}^{2}\;\;\;\;\;\frac{p_{⊥\psi }^{\left(-\right)}}{R_{⊥\psi }^{A}}=\frac{p_{⊥⊥}^{\left(-\right)}}{R_{⊥⊥}^{A}}\cos^{2}\psi +\frac{p_{⊥\|}^{\left(-\right)}}{R_{⊥\|}^{A}}\sin^{2}\psi$
	$\cos^{2}\psi =\frac{\tau_{T}^{2}}{\tau_{T}^{2}+\tau_{L}^{2}}\;\;\;\sin^{2}\psi =1-\cos^{2}\psi$
LaRC05	${\left(\frac{\tau_{T}}{R_{⊥⊥}^{A}-p_{⊥⊥}^{\left(-\right)}\sigma_{N}}\right)}^{2}+{\left(\frac{\tau_{L}}{R_{⊥\|}^{A}-p_{⊥\|}^{\left(-\right)}\sigma_{N}}\right)}^{2}=1$

**Table 4:** Failure criteria for fiber-dominated compressive failure.

Theory	Description
Maximum stress	$\frac{\left|\sigma_{11}\right|}{X_{C}}=1$
Fiber-kinking theory	$F_{M}\left(\sigma_{22}^{\varphi },\sigma_{33}^{\varphi },\tau_{23}^{\varphi },\tau_{31}^{\varphi },\tau_{21}^{\varphi }\right)=1$
	$F_{M}\left(\sigma_{N}^{\varphi },\tau_{T}^{\varphi },\tau_{L}^{\varphi }\right)=1$

**Table 5:** Input parameters for different failure criteria.

Theory	Input Parameters	Comments
Hashin	$S_{23}=55$	$S_{23}=Y_{T}$
Camanho	$\alpha_{1}=3.31\times 10^{-4}\;\;\alpha_{2}=1.49\times 10^{-4}$	Equation (14)
	$\alpha_{3}^{c}=1.40\times 10^{-2}\;\;\alpha_{32}^{c}=6.92\times 10^{-6}$	Assume $Y_{BC}=3Y_{C}$
	$\varphi_{0}=3.81{}^{\circ}$	Equation (20)
Puck	$p_{⊥⊥}^{\left(-\right)}=0.250\;\;R_{⊥⊥}^{A}=84.0$	Fracture plane angle 51°
	$p_{⊥\|}^{\left(-\right)}=0.243\;\;R_{⊥\|}^{A}=81.8$	Equation (18)
LaRC05	$p_{⊥⊥}^{\left(-\right)}=0.176\;\;R_{⊥⊥}^{A}=88.2$	Fracture plane angle 51°
	$p_{⊥\|}^{\left(-\right)}=0.243\;\;R_{⊥\|}^{A}=81.8$	Equation (18)
	$\varphi_{0}=3.71{}^{\circ}$	Equation (20)

## Data Availability

The data presented in this study are available on request from the corresponding author.
